# Multiple Sclerosis Relapse Activity After Ozanimod Discontinuation in DAYBREAK Trial Participants

**DOI:** 10.1002/acn3.70366

**Published:** 2026-03-27

**Authors:** Ralf Gold, Krzysztof W. Selmaj, Regina Berkovich, Jeffrey A. Cohen, Giancarlo Comi, Eva K. Havrdová, James K. Sheffield, Hetal Desai, Chun‐Yen Cheng, Jon V. Riolo, Andrew Thorpe, Erik DeBoer, Bruce A. C. Cree

**Affiliations:** ^1^ Neurologische Universitätsklinik St. Josef Hospital Bochum Germany; ^2^ Center of Neurology Lodz Poland; ^3^ Department of Neurology University of Warmia & Mazury Olsztyn Poland; ^4^ Medical Imaging Center of Southern California Santa Monica California USA; ^5^ Mellen Center for Multiple Sclerosis Treatment and Research Cleveland Clinic Cleveland Ohio USA; ^6^ Vita‐Salute San Raffaele University and Casa di Cura Igea Milan Italy; ^7^ Department of Neurology and Center for Clinical Neuroscience First Medical Faculty and General University Hospital, Charles University Prague Czech Republic; ^8^ Bristol Myers Squibb Princeton New Jersey USA; ^9^ Weill Institute for Neurosciences, Department of Neurology University of California, San Francisco San Francisco California USA

**Keywords:** discontinuation, disease‐modifying therapy, multiple sclerosis, ozanimod, rebound, relapse

## Abstract

**Objective:**

Return of disease activity is expected when patients discontinue disease‐modifying therapy (DMT) for multiple sclerosis (MS). Some MS DMTs are associated with higher‐than‐expected disease activity (rebound) after discontinuation. This exploratory analysis assessed disease activity and risk of rebound after ozanimod discontinuation.

**Methods:**

DAYBREAK (NCT02576717; October 2015–April 2023) was a single‐arm, open‐label extension trial of ozanimod 0.92 mg/d in participants with relapsing MS who completed phase 1–3 ozanimod trials. Two protocol amendments increased the required safety follow‐up from an initial 28 days to 75 and then 90 days. Confirmed post‐treatment relapses were characterized.

**Results:**

Of 2494 participants, 1679 participants had ≥ 1 day of follow‐up and did not initiate commercial ozanimod in ≤ 14 days of exiting the trial; 30.6% had ≤ 60 days of post‐treatment follow‐up. Fifty‐five participants (3.3%) had known post‐treatment relapse. Fifty‐four were not taking an MS DMT when they relapsed; one relapsed soon after starting fingolimod. Relapses were associated with Expanded Disability Status Scale (EDSS) score increases of 0–3 points (median 1 point); the participant with the 3‐point increase had a moderate relapse and complete recovery in 23 days. Forty‐two participants (76.4%) fully recovered, 11 (20.0%) partially recovered, and 2 (3.6%) did not recover. Only one (1.8%) relapse was considered severe by the investigator: EDSS increased from 7.5 to 8.0, and the participant partially recovered within 36 days.

**Interpretation:**

Most participants did not relapse within 90 days following ozanimod discontinuation. Post‐treatment relapse cases suggestive of a rebound effect were not observed.

## Graphical abstract

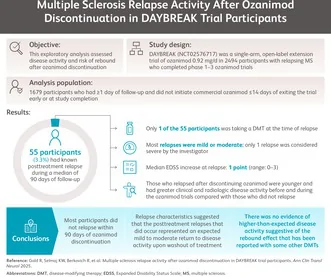

## 1 Introduction

Multiple sclerosis (MS) disease‐modifying therapies (DMTs) effectively reduce clinical relapses and lesion activity on magnetic resonance imaging (MRI) [1]. In clinical practice, there are many reasons why patients with MS discontinue DMTs, including family planning/pregnancy, insufficient efficacy, adverse effects, comorbid conditions, and patient preference [2]. Return of previous disease activity, including relapse, is expected after MS DMTs are discontinued, if patients do not promptly initiate a new DMT [3, 4].

Less commonly, some patients experience higher‐than‐expected disease activity, described as “rebound,” after discontinuation of natalizumab and some sphingosine 1‐phosphate (S1P) receptor modulators, as well as other MS DMTs [2, 5, 6, 7, 8, 9, 10]. The potential for rebound can be an important consideration when initiating MS DMTs. While the typical clinical presentation of rebound is characterized by fulminant disease [2], there is no standardized, universally accepted definition of the level and type of disease activity that constitutes rebound. Widely varying definitions and criteria were used in previous publications of S1P receptor modulators evaluating a potential rebound effect (Table S1) [5, 6, 11, 12, 13, 14, 15, 16, 17].

Ozanimod, a selective S1P receptor 1 and 5 modulator, is a DMT approved in multiple countries for the treatment of adults with either relapsing forms of multiple sclerosis (RMS) or moderately to severely active ulcerative colitis [18, 19]. This exploratory, post hoc analysis assesses return of clinical disease activity and risk of rebound after ozanimod discontinuation among adults with RMS who exited the DAYBREAK open‐label extension trial.

## 2 Methods

### 2.1 Study Design

DAYBREAK (NCT02576717) was a phase 3, single‐arm, open‐label extension trial of ozanimod 0.92 mg/d that enrolled adults with RMS who previously completed phase 1–3 “parent” trials of ozanimod. DAYBREAK was conducted in 25 countries in Europe and North America, plus South Africa and New Zealand, with about 90% of participants coming from Eastern Europe. An interim analysis with detailed methodology was reported [20]. The final safety and efficacy results are available on Clinicaltrials.gov and have been published separately [21].

### 2.2 Ethical Considerations

DAYBREAK and its parent trials received approval from the institutional review board or ethics committee at each site. The study was conducted in accordance with the ethical principles originating in the Declaration of Helsinki, and participants (or their legally acceptable representatives) provided written informed consent before participating.

### 2.3 Study Population

The phase 1–3 parent trials of ozanimod enrolled adults aged 18 to 55 years with RMS. Additional key inclusion criteria in the phase 2 and 3 trials included lesions consistent with MS on MRI; 1 or more relapses in the past 12 months or 1 or more relapses in the past 24 months plus at least 1 gadolinium‐enhancing (GdE) lesion on brain MRI; and no relapses or corticosteroid or adrenocorticotropic hormone use in the past month [22, 23, 24]. Participants in the phase 2 and 3 trials also had to have an Expanded Disability Status Scale (EDSS) score of 0 to 5 [22, 23, 24]. The phase 1 trial had similar inclusion criteria except that participants had to have a relapsing clinical course without specific requirements related to number of relapses in the preceding months and they had to have an EDSS score of 0 to 6. The participants who completed one of the phase 1–3 trials were eligible to enroll in DAYBREAK [20].

### 2.4 Post‐Treatment Follow‐Up Duration and Analysis Group

At study initiation on October 16, 2015, protocol‐defined safety follow‐up after ozanimod discontinuation was 28 days. It was increased to 75 days in 2018, and 90 days from 2019 through study completion (database lock: April 7, 2023). Follow‐up was not required past 90 days; however, a ±10‐day window was permitted for the 90‐day follow‐up visit. After the study, follow‐up visits were not required if commercial ozanimod was started within 14 days of study drug discontinuation. (Commercial ozanimod was not available to all participants who discontinued the study.) Use of other MS DMTs was permitted after the 28‐day follow‐up visit. The analysis population for assessment of post‐treatment rebound consisted of DAYBREAK participants with ≥ 1 day of post‐treatment safety follow‐up who did not, or could not, transition to commercial ozanimod within 14 days.

### 2.5 Outcomes

Study participants were instructed to contact their study site should they experience new or worsening neurological symptoms to schedule a study visit for relapse assessment. The post‐treatment safety period included protocol‐required in‐person (or telephone, if in‐person was not feasible) contact with the investigator for final assessment of any adverse events. Confirmed MS relapses after permanent ozanimod discontinuation were assessed and described by the treating investigator for timing, severity, duration, change in EDSS score, and recovery. Post‐treatment relapses were identified based on protocol‐required collection of data during unscheduled relapse visits, which were to be held as soon as possible and ideally within 7 days of symptom onset, during the entire DAYBREAK study, including the safety follow‐up period. Relapses were defined as any occurrence of new or worsening neurologic symptoms that lasted > 24 h, were attributable to MS with no confounding clinical factors, and were immediately preceded by a relatively stable or improving neurologic state for at least 30 days. The new/worsening neurologic symptoms had to be accompanied by objective neurologic worsening (i.e., at least 0.5 points on the EDSS, or 2 points on a Functional System [FS] score in a function corresponding to the participant's symptoms, or 1 point on ≥ 2 relevant FS scores relative to the last EDSS or FS score that was not during a relapse). For this analysis, we report confirmed relapses that met the objective criteria as well as confirmed plus unconfirmed relapses. Participants were advised to notify the treating investigator within 48 h of symptom onset, and the protocol required the investigator to conduct a telephone interview and arrange the unscheduled relapse visit. MRIs were not required during unscheduled visits for MS relapse.

While there is no universally accepted definition of rebound or parameters for defining severe exacerbation (see Table S1 for proposed rebound definitions), for this analysis, potential rebound was defined as a severe exacerbation of disease, as identified by the investigator, or a severe persistent increase in disability. Available clinical information is presented for participants with objectively confirmed relapse after permanent ozanimod discontinuation to further characterize these episodes.

### 2.6 Data and Statistical Analysis

Relapse timing and characteristics were described using descriptive statistics. Demographics and clinical characteristics were described using descriptive statistics and were compared for subgroups of participants with and without post‐treatment relapse. Nominal *P* values were generated using the Wilcoxon rank‐sum test for continuous variables and Fisher's exact test for categorical variables. Stepwise multivariable logistic regression was used to identify potential predictors of post‐treatment relapse.

## 3 Results

### 3.1 Participant Disposition

There were 2494 participants in DAYBREAK with up to 117.2 months (9.8 years) of cumulative ozanimod exposure in the parent trials and extension. Nearly 80% of participants (1950/2494) who entered DAYBREAK completed the study, and 544 discontinued early. Of the overall population, 67% (1679/2494) were included in the rebound analysis population after excluding participants with no protocol‐required safety follow‐up and those who initiated commercial ozanimod within 14 days of terminating study drug (Figure 1). Of those 1679 participants, 23.9% had ≤ 28 days of post‐treatment follow‐up, 6.7% had 29–60 days, 28.1% had 61–90 days, and 41.3% had > 90 days of post‐treatment follow‐up (median: 90 days; range: 1–374 days; Figure S1). Three participants initiated commercial ozanimod during the safety follow‐up, but after the protocol‐defined 14‐day window. Participants who were excluded because they were lost to follow‐up had demographics and clinical characteristics comparable to those of the analysis population (Supplemental Table S2).

**FIGURE 1:**
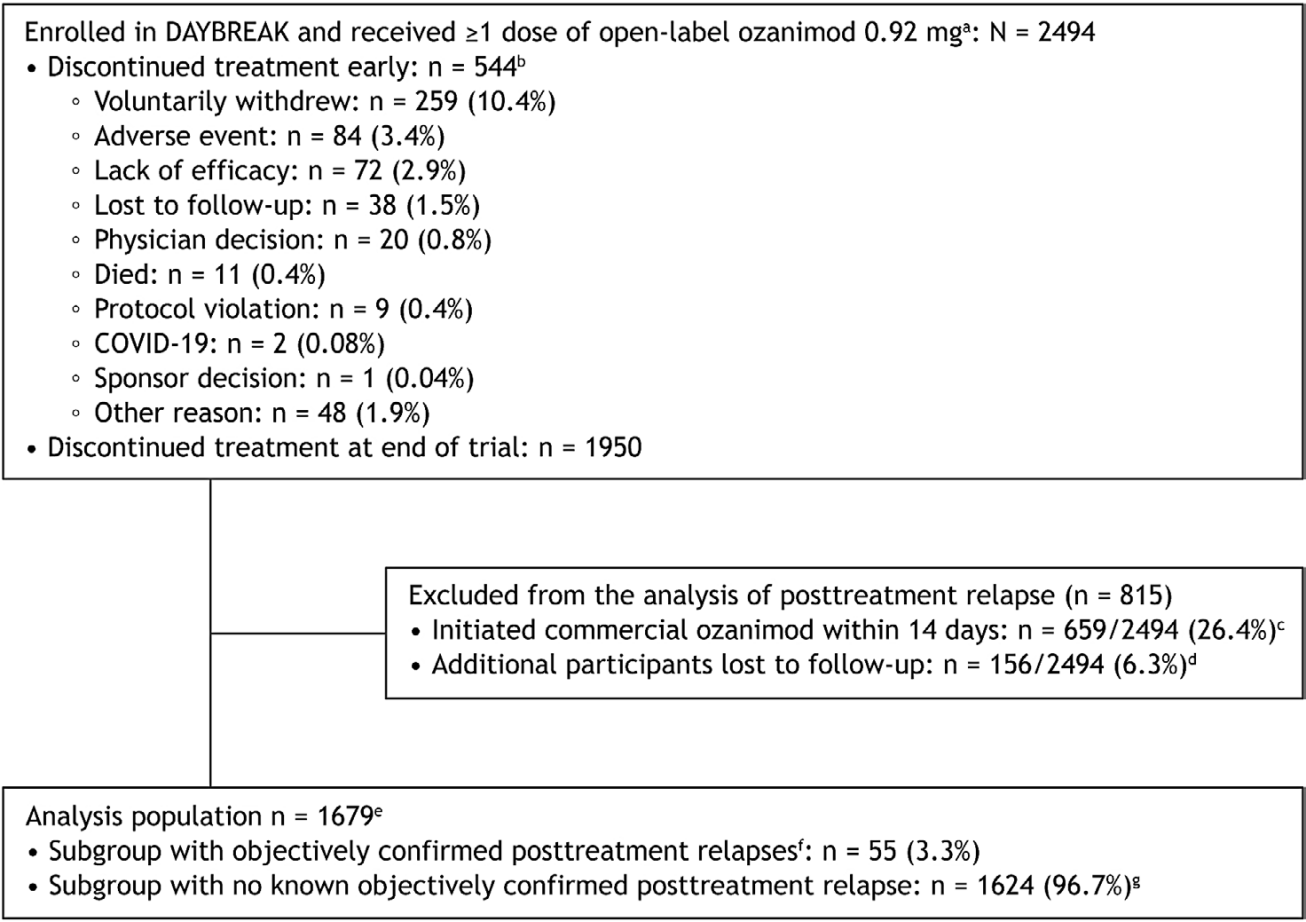
DAYBREAK participant flow. ^a^Mean ozanimod exposure during DAYBREAK was 60.9 months (range: 0.03–81.5 months). Cumulative ozanimod exposure in the parent trials and DAYBREAK was a mean of 74.8 months (range 0.03–117.2 months). ^b^Among the 544 who discontinued early, 29 did so due to pregnancy, and 87 did so for family planning purposes. ^c^DAYBREAK participants who transitioned to commercial ozanimod within 14 days of discontinuing study drug were not required to attend safety follow‐up visits after their end of treatment visit. Of the 659 participants who were excluded for starting commercial ozanimod within 14 days, 367 also had no safety follow‐up. Three participants initiated commercial ozanimod during the safety follow‐up, but after the protocol‐defined 14‐day window, and were not excluded from the analysis. It should be noted that 1577 (93.9%) of DAYBREAK participants were from Eastern Europe, where ozanimod has not been commercialized with the exception of Poland. Therefore, a majority of the participants did not have the option of continuing ozanimod after completing or withdrawing from the trial. ^d^Due to logistical challenges in ensuring continuity of care, the sponsor expedited closure of study sites in Russia by June 2022 and transitioned all affected DAYBREAK participants (*n* = 287) to standard of care; day 28 and day 90 follow‐up visits were not performed for these participants. In addition, 6 sites in Ukraine faced operational challenges due to the Russia‐Ukraine war, and 77 participants from Ukraine did not complete the trial. ^e^Of the 1679 participants analyzed, 78 initiated another MS DMT during the safety follow‐up period; only 1 participant relapsed while taking another DMT. ^f^Mean ozanimod exposure during DAYBREAK among the 55 participants with post‐treatment relapse was 61.8 months (range: 25.5–70.9 months). ^g^Thirteen patients were evaluated for suspected relapse but did not meet the protocol‐required criteria for objective neurologic worsening (i.e., at least 0.5 points on the Expanded Disability Status Scale [EDSS], or 2 points on a Functional System [FS] score in a function corresponding to the participant's symptoms, or 1 point on ≥ 2 relevant FS scores relative to the last EDSS or FS score that was not during a relapse). DMT, disease‐modifying therapy.

### 3.2 Incidence and Timing of Post‐Treatment Relapse

Fifty‐five (3.3%) of the 1679 participants in the analysis population had objectively confirmed relapses after ozanimod discontinuation. An additional 13 participants were evaluated for a suspected relapse but did not meet the objective criteria; thus, 68/1679 (4.1%) had confirmed or unconfirmed relapse.

Among the participants with confirmed relapse, time to onset of relapse after permanent discontinuation was 3–141 days (median 61 days) post‐treatment. Relapses occurred in 5/1679 (0.3%) participants during the first 28 days post‐treatment (Figure 2). In addition, 22 participants relapsed 29–60 days after ozanimod discontinuation, representing 1.7% of the 1277 participants with follow‐up durations of > 28 days. Another 26 participants relapsed 61–90 days after ozanimod discontinuation, representing 2.2% of the 1165 with follow‐up of > 60 days. Two additional participants out of 694 with post‐treatment follow‐up > 90 days (0.3%) relapsed > 90 days after stopping ozanimod. One of those participants relapsed at 93 days, which was within the 10‐day follow‐up window, and the other, who relapsed after 141 days, was nonadherent to treatment due to COVID‐19 travel restrictions. Results were similar when relapses were analyzed using follow‐up categories of 1–28 days, 29–65 days, 66–85 days, and ≥ 85 days (Supplemental Figure S2).

**FIGURE 2:**
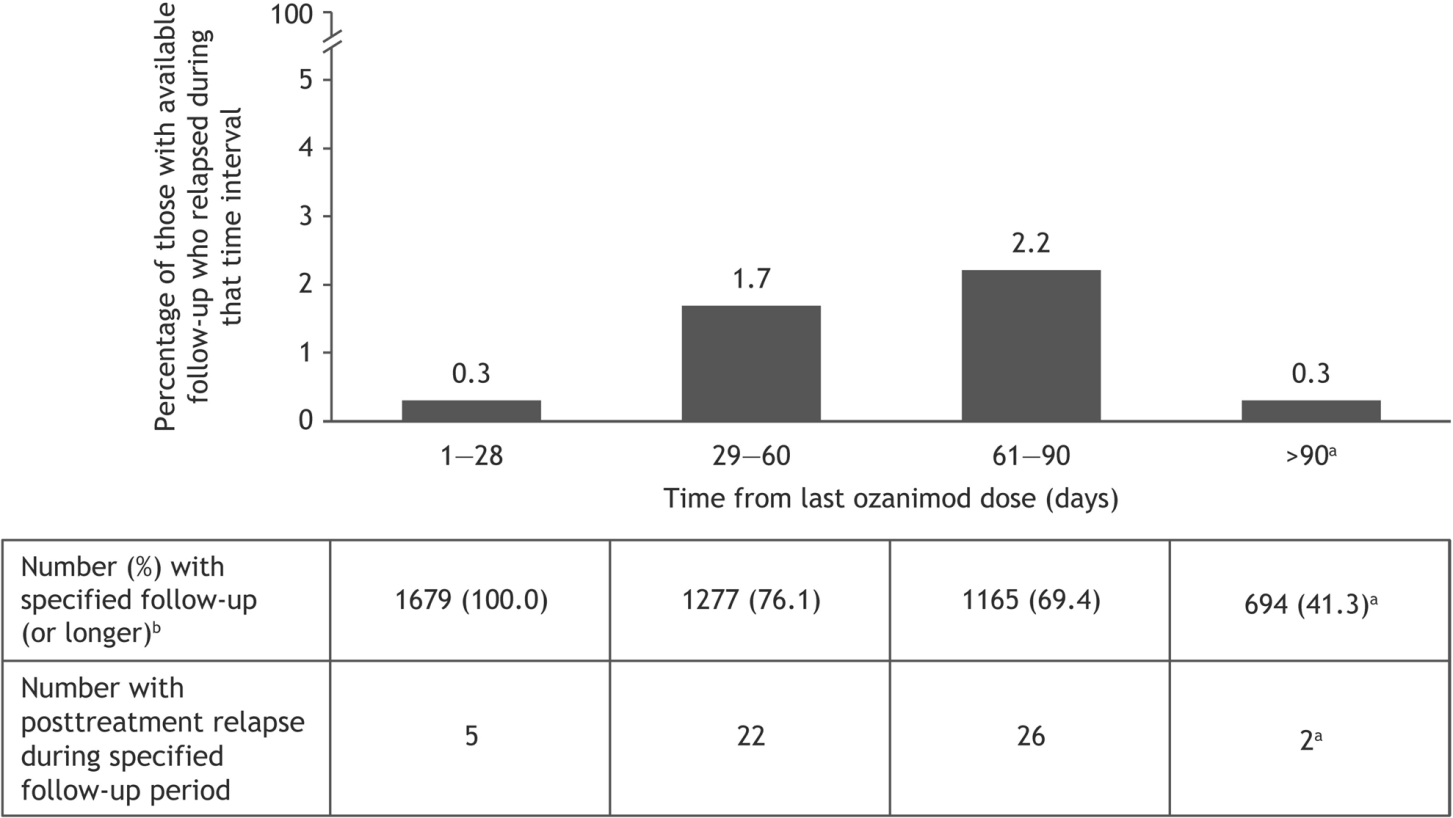
Timing of post‐treatment disease reactivation (relapse) (*n* = 55) after ozanimod discontinuation. ^a^Participants were not required to have more than 90 days of follow‐up, but a ±10‐day window was permitted for the 90‐day follow‐up visit. One participant who relapsed after 93 days was within the protocol‐defined ±10 days follow‐up window. The other, who relapsed after 141 days, was nonadherent to treatment due to COVID‐19 travel restrictions. ^b^Excludes participants with < 1 day of follow‐up and those who switched to commercial ozanimod.

### 3.3 Characterization of Post‐Treatment Relapses

Of the 1679 participants analyzed, 78 (4.6%) switched to another MS DMT after discontinuing ozanimod. The median time from discontinuing ozanimod to starting another DMT was 52 days (range: 5–148 days). Five of the 78 participants relapsed before they started the next DMT. One of the 78 participants (1.3%) relapsed after starting a new DMT: This participant started fingolimod 70 days after discontinuing ozanimod and then relapsed at day 83 (i.e., 13 days after initiating fingolimod). No other participants with post‐treatment relapse were taking any MS DMT at the time of relapse.

Of the 55 participants who relapsed, 44 had discontinued ozanimod at the end of the trial, and 11 had discontinued ozanimod early. Reasons for early discontinuation among those 11 participants were voluntary withdrawal (*n* = 7), adverse event (*n* = 1), lack of efficacy (*n* = 1), protocol violation (*n* = 1), and lost to follow‐up (*n* = 1).

Nearly all post‐treatment relapses were mild or moderate based on investigator‐determined severity. Only one participant (1.8%) had a relapse identified by the investigator as severe (Table 1), and we concluded that this case did not represent higher‐than‐expected disease activity after ozanimod discontinuation based on the participant's disease activity history. Before the parent‐trial baseline, this participant was not on any MS DMT and had experienced 2 relapses in the 2 years before the parent‐trial baseline. At parent‐trial baseline, this female participant was 44 years of age, had no GdE, 70 T2 lesions (7.97 cc volume) on MRI, and an EDSS score of 5.0. This participant experienced 3 relapses while taking ozanimod during the trials, including 1 moderate relapse in the parent trial, 1 moderate relapse during DAYBREAK, and a relapse that was considered severe by the investigator that occurred about 10 months before the end of ozanimod treatment and about 13 months before the post‐treatment relapse. This participant was wheelchair bound at the time of ozanimod discontinuation (EDSS 7.5) and during the post‐treatment relapse (EDSS 8.0). The participant was not hospitalized for the post‐treatment relapse, and no MRI was ordered by the investigator. Partial recovery from relapse occurred within 36 days.

**TABLE 1:** Characteristics of relapses after ozanimod discontinuation (*n* = 55).

Post‐treatment relapse characteristics	Results	Selected case details
Severity^a^ *n* (%)		
Mild	20 (36.4)	
Moderate	34 (61.8)	
Severe	1 (1.8)	Relapse 84 days after ozanimod discontinuation with 0.5‐point increase in EDSS (from 7.5 to 8.0), and partial recovery within 36 days. The participant was not hospitalized and no MRI was ordered by the investigator. The participant was wheelchair bound at the time of ozanimod discontinuation and during relapse. The participant also experienced a severe relapse, as defined by the investigator, in the prior year while taking ozanimod
Duration, days, median (range)	22 (4–74)	
Hospitalization, *n* (%)	14 (25.5)	Eleven were from Eastern European countries (Poland *n* = 3, Ukraine *n* = 3, Belarus *n* = 3, Bulgaria *n* = 1, Bosnia and Herzegovina *n* = 1) and 3 were from South Africa
Occurrence during MS DMT use, *n* (%)^b^	1 (1.8)	Started fingolimod 70 days after discontinuing ozanimod and relapsed at day 83
ALC at visit prior to relapse, × 10^9^/L, median (range)	0.9 (0.2–1.8)	
Change in EDSS relative to last assessment before relapse, median (range)^c^	1 (0–3)	
EDSS category change relative to last assessment, *n* (%)^c^		
0	3 (5.5)	No EDSS increase, but increases in other functional scales were reported.
0.5	22 (40.0)	
1.0	14 (25.5)	
1.5	8 (14.5)	
2.0	4 (7.3)	All moderate relapses, with EDSS increases from: 4.5 to 6.5 with complete recovery 1.5 to 3.5 with complete recovery 1.5 to 3.5 with partial recovery; final EDSS: 2.0 2.0 to 4.0 with partial recovery; final EDSS: 4.0
2.5	2 (3.6)	Moderate relapse with EDSS increase from 1.5 to 4.0 with complete recovery Moderate relapse with EDSS increase from 3.0 to 5.5 with partial recovery; final EDSS: 5.5
3.0	1 (1.8)	Moderate relapse with EDSS increase from 2.5 to 5.5 and complete recovery in 23 days
Investigator‐determined recovery, *n* (%)^a^		
None	2 (3.6)	Both moderate relapses with EDSS increases of 0.5 and 1.0
Partial	11 (20.0)	
Complete	42 (76.4)	

*Note:* None of the participants had MRIs performed at relapse.

Abbreviations: ALC, absolute lymphocyte count; DMT, disease‐modifying therapy; EDSS, Expanded Disability Status Scale; MRI, magnetic resonance imaging.

^a^ Relapse severity and recovery from relapse were based on the investigator's determination; investigators were not provided with specific definitions of these categories.

^b^ There were 78 participants who initiated another MS DMT during the safety follow‐up period.

^c^ One participant with a confirmed relapse did not have a protocol‐required EDSS documented at the time of relapse by the investigator.

Among those in the analysis population with post‐treatment relapse and EDSS data available (*n* = 54), EDSS scores increased by 0 to 3 points (median 1 point) at the post‐treatment relapse, relative to the last assessment. Details of 7 relapses associated with EDSS increases of ≥ 2 points are provided in Table 1. Based on investigator assessment at the time of last safety follow‐up, 76.4% of the 55 participants with post‐treatment relapse fully recovered, 20.0% partially recovered, and 2 participants (3.6%) did not recover. Four of the 7 participants with EDSS increases ≥ 2 points made a complete recovery, and the remaining 3 had partial recoveries by data cutoff.

### 3.4 Characteristics of Participants Who Experienced Post‐Treatment Relapse

The subgroup with post‐treatment relapse was younger, had more GdE and T2 lesions at baseline, and more relapses during the parent and DAYBREAK extension trials, including in the 12 months before discontinuation, than those who did not relapse after treatment (Table 2). Multivariable analysis found age at MS diagnosis, number of relapses during the OLE, and T2 lesion count at parent trial baseline to be nominally significantly associated with post‐treatment relapse (Table 3).

**TABLE 2:** Baseline demographic and disease characteristics of those with and without post‐treatment relapse.

Participant characteristics	Subgroup with post‐treatment relapse (*n* = 55)	Subgroup without post‐treatment relapse (*n* = 1624)	Nominal *p* value (subgroups comparison)^a^
Age at OLE consent, year, mean (SE) [range]	33.2 (1.10) [19–51]	36.8 (0.22) [19–57]	0.0047
Sex, female, *n* (%)	42 (76.4)	1078 (66.4)	0.1457
Race, White, *n* (%)	54 (98.2)	1618 (99.6)	0.2083
Region, Eastern Europe, *n* (%)	50 (90.9)	1527 (94.0)	0.3794
BMI ≥ 30 kg/m^2^, *n* (%)	2 (3.6)	150 (9.2)	0.2281
Active smoker at OLE baseline, *n* (%)	9 (16.4)	333 (20.5)^b^	0.6090
MS DMT use prior to parent trial enrollment, *n* (%)	21 (38.2)	443 (27.3)	0.0907
EDSS at OLE baseline, median (range), points	3.0 (0–6)	2.5 (0–7.5)	0.1252
Age at MS symptom onset, years, mean (SE) [range]	24.1 (0.93) [10–42]	28.7 (0.22) [9–53]^c^	0.0001
Age at MS diagnosis, years, mean (SE) [range]	27.1 (1.05) [14–46]	32.0 (0.22) [13–55]^c^	0.0001
Number of relapses in 12 months before parent trial screening, mean (SE) [range]	1.3 (0.09) [0–3]	1.3 (0.02) [0–5]	0.9660
Number of relapses in 24 months before parent trial screening, mean (SE) [range]	1.7 (0.11) [1–5]	1.8 (0.02) [0–14]^d^	0.6762
GdE lesion count at parent trial baseline, mean (SE) [range]	3.2 (0.63) [0–19]	1.8 (0.08) [0–26]^e^	0.0039
T2 lesion count at parent trial baseline, mean (SE) [range]	73.2 (5.51) [11–197]	53.0 (0.91) [0–222]^e^	0.0001
Number of relapses during phase 2 or 3 parent trial, mean (SE) [range]	0.8 (0.17) [0–5]	0.4 (0.02) [0–7]^c^	0.0240
Number of relapses during treatment in the OLE, mean (SE) [range]	2.3 (0.37) [0–11]	0.8 (0.04) [0–11]	< 0.0001
Participants with relapse in the 12 months prior to ozanimod discontinuation, *n* (%)	17 (30.9)	230 (14.2)	0.0016

Abbreviations: BMI, body mass index; DMT, disease‐modifying therapy; EDSS, Expanded Disability Status Scale; GdE, gadolinium‐enhancing; MS, multiple sclerosis; OLE, open‐label extension; SE, standard error.

^a^ Nominal *P* values are based on the Wilcoxon rank‐sum test for the continuous variables and Fisher's exact test for categorical variables.

^b^ *n* = 1619.

^c^ *n* = 1617.

^d^ *n* = 1624.

^e^ *n* = 1616.

**TABLE 3:** Stepwise multivariable logistic regression model.

Characteristic^a^	Estimate (95% CI)	OR (95% CI)	Nominal *p* value
Intercept	−2.26 (−3.45, −1.08)		0.0002
Age at MS diagnosis, years	−0.07 (−0.10, −0.03)	0.94 (0.90, 0.97)	0.0005
Number of relapses during treatment in the OLE	0.30 (0.19, 0.41)	1.35 (1.21, 1.50)	< 0.0001
T2 lesion count at parent trial baseline	0.0070 (0.0005, 0.0135)	1.0070 (1.0005, 1.0136)	0.0359

Abbreviations: AIC, Akaike information criterion; CI, confidence interval; MS, multiple sclerosis; OLE, open‐label extension; OR, odds ratio.

^a^ Nominal *P* value of the likelihood‐ratio test = < 0.0001; AIC = 438.424; adjusted R‐square = 0.0291.

## 4 Discussion

Discontinuing any MS DMT eventually leads to loss of therapeutic effect; however, some DMTs, particularly fingolimod and natalizumab, were associated with cases of higher‐than‐expected disease activity after discontinuation, which was described as “rebound.” [5, 6, 8] An understanding of the likelihood and expected timing of severe disease worsening after DMT cessation can help guide patient expectations and clinical decision‐making. In this analysis of adults with RMS who participated in the DAYBREAK open‐label extension trial of ozanimod 0.92 mg, 3.3% of participants experienced clinical relapse in the follow‐up period (median 3 months) after discontinuing ozanimod. Relapse characteristics suggested that these cases represented a mild to moderate return to disease activity upon treatment discontinuation.

In this manuscript, potential rebound was defined as severe exacerbation of disease as identified by the investigator or severe persistent increase in disability. Clinical information was provided for context. One case of relapse was deemed severe by the investigator; this participant's EDSS increased from 7.5 to 8.0, and the investigator considered the participant to be partially recovered by the end of follow‐up, although details of what constituted partially recovered were not available. Three participants with relapses of moderate severity had EDSS increases ≥ 2 points; they partially recovered by the end of the short follow‐up period. It is our opinion that none of these cases constitute higher‐than‐expected disease activity in participants with untreated RMS, and therefore, we do not consider there to be any evidence of rebound following ozanimod discontinuation in this data set. Furthermore, ozanimod was first approved for treatment of RMS in 2020, and no cases of rebound following ozanimod discontinuation have been reported in the medical literature.

Several definitions of rebound after DMT discontinuation were used in previous analyses of S1P modulators (Table S1). Unfortunately, we were unable to apply many of those definitions to the current data set because: (a) MRI data were not collected after discontinuation of ozanimod, (b) the duration of post‐treatment follow‐up in DAYBREAK was short, and (c) available data regarding pre‐treatment disease history prior to parent‐trial enrollment were limited. The most comparable previous analysis with an applicable definition was the assessment of “higher‐than‐expected disease” among participants in the fingolimod phase 3 FREEDOMS and FREEDOMS II trials [6]. Post‐treatment follow‐up duration was 3 to 7 months in the FREEDOMS trials, which is longer than the 28 to 90 days specified in the DAYBREAK trial protocol for ozanimod. Higher‐than‐expected disease activity was defined in the analysis of the FREEDOMS trials as relapse with either symptoms considered severe by the investigator; hospitalization; incomplete recovery; or EDSS increase of ≥ 3 points if prior score of 0, ≥ 2 points if previously 1–5, and ≥ 1 point if previously > 5 compared to last EDSS on treatment or within 30 days of discontinuation [6]. In that analysis, rates of higher‐than‐expected disease after discontinuing fingolimod 0.5 mg were 4.0% in FREEDOMS and 3.5% in FREEDOMS II, and 4.4% and 4.1% in the placebo groups of those trials, respectively [6]. Authors of that analysis attributed these cases to the underlying variability of RMS rather than to a rebound effect [6]. Fingolimod was the first S1P modulator developed for MS; thus, the authors may have overlooked the severity of these relapses since little was known about the rebound effect at that point. If the same definition is applied to participants who discontinued ozanimod during or after the DAYBREAK trial, 1.4% of the analysis population meets criteria for “higher‐than‐expected disease.” Of the 24 participants meeting ≥ 1 of those criteria, 14 were hospitalized during relapse, and 6 of those hospitalized did not meet any of the other criteria. It should be noted that 11 of the 14 participants who were hospitalized were from Eastern Europe, where it is common to treat patients with intravenous steroid treatment for MS relapse as inpatients, regardless of relapse severity. Although FREEDOMS was conducted internationally in 22 countries [25], FREEDOMS II participants were largely from the United States [26] where less severe relapses are commonly treated in the outpatient setting.

As an additional check, we compared the change in EDSS scores at post‐treatment relapse relative to the assessment before relapse in DAYBREAK with historical, previously published relapse data in a population of untreated patients [27]. While we acknowledge the limitations of cross‐trial comparisons with different patient populations, non‐zero changes in EDSS during relapse were similar between the 2 data sets (Supplemental Figure S3) and were not statistically significantly different when compared using nonparametric tests. Thus, in an informal comparison, we found no evidence of a greater increase in disability during relapse among patients discontinuing ozanimod than would be expected during relapse in an untreated population with MS, supporting our conclusion that there was no evidence of a rebound effect following ozanimod discontinuation in this post hoc analysis of DAYBREAK participants.

In this analysis, those who relapsed after discontinuing ozanimod were younger and had greater clinical and radiologic disease activity before and during the ozanimod trials compared with those who did not relapse during up to 3 months of follow‐up. This is consistent with previous analyses of other MS DMTs that showed that patients with higher relapse rates prior to or during DMT use have a greater risk of relapse after discontinuing a DMT [15, 28, 29]. Our findings are also consistent with the fact that relapse rates among MS patients in general peak around ages 20 to 30 years and then decline with age [30]. Caution may be needed when younger patients with more active disease discontinue ozanimod without starting another MS DMT, since a return to disease activity may be anticipated.

The timing of disease activity return after treatment discontinuation is linked to the specific pharmacokinetic profile and mechanism of the DMT. Ozanimod is a selective S1P receptor 1 and 5 modulator that reversibly blocks the egress of lymphocytes from lymphoid tissues, thereby decreasing blood lymphocyte counts [18, 19, 31]. The therapeutic effect of ozanimod in MS is thought to involve the reduction in lymphocyte migration into the CNS [18]. Ozanimod and its major active metabolite elimination half‐lives are 21 h and approximately 11 days, respectively [18, 19]. After ozanimod discontinuation, lymphocyte levels in the blood return to normal after a median of 30 days, with 80% to 90% of patients in the normal range within 3 months [18, 19]. Although the relationship between repopulation of lymphocytes in peripheral blood and MS disease activity is not fully understood, the increase in relapses observed at around 2 to 3 months (median 61 days) post‐discontinuation corresponds to the period of lymphocyte release into the circulation after ozanimod elimination.

Although return of disease activity is expected after discontinuation of any MS DMT, rebound effects were reported for natalizumab and fingolimod in particular. Fingolimod has broader S1P receptor binding than ozanimod (it binds to S1P 1, 3, 4, and 5 receptors) but a similar mechanism of action involving sequestration of lymphocytes [32]. Fingolimod reduces absolute lymphocyte count more than ozanimod [33]. Fingolimod has a half‐life of 6 to 9 days, and return of disease activity commonly occurs about 2 to 4 months after fingolimod discontinuation [5, 13, 14]. As noted, rates of “higher‐than‐expected disease” after discontinuing fingolimod 0.5 mg were 4.0% and 3.5% among phase 3 FREEDOMS and FREEDOMS II trial participants, respectively, but were similar at 4.4% and 4.1% in the placebo group [6]. Rebound rates in observational studies of fingolimod ranged from 5% to 42.8% in analyses with varying definitions of rebound and post‐treatment follow‐up durations of < 1 week to 47 months [5, 11, 12, 13, 14, 15, 16, 17]. Cases of tumefactive demyelinating lesions concurrent with severe relapse were reported following cessation of fingolimod [34, 35]. Although MRI was not performed in DAYBREAK at post‐treatment relapse, the development of tumefactive demyelinating lesions was never reported either during or after ozanimod treatment.

Natalizumab, an adhesion‐molecule inhibitor that binds to the alpha4‐subunit of integrins present on most human leukocytes, prevents leukocyte migration across endothelium into inflamed parenchymal tissue [36]. Cessation of natalizumab allows resumption of leukocyte migration across endothelial membranes, including the blood–brain barrier [37, 38]. Natalizumab has a mean half‐life of about 11 days [36]. Clinical disease activity returns in the first 1 to 3 months after discontinuation of natalizumab [28, 39] and reaches baseline levels at 4 to 7 months [37]. Rebound rates following natalizumab discontinuation were 8% to 22% in a systematic literature review of studies with varying definitions and follow‐up durations [8]. Severe cases with multifocal neurologic worsening and large increases in T2 and/or GdE lesions despite initiation of an alternate MS DMT were reported [38, 40].

Comparisons of rebound effects for different MS DMTs should be made with caution. Cross‐study comparisons of rebound rates are limited by inconsistent rebound definitions, varying follow‐up durations, and differences in analysis populations. For example, observational data are more likely to include patients who discontinued treatment due to lack of efficacy and who, therefore, may already have been experiencing disease activity prior to MS DMT withdrawal, whereas most patients in our analysis discontinued ozanimod due to completion of the trial. Definitions of rebound that rely on comparison with pretreatment disease activity are potentially confounded by factors such as prior MS DMT use before study enrollment and the duration of time passed since study enrollment.

This study has important limitations that must be acknowledged. DAYBREAK was not designed to assess post‐treatment relapse or to compare those with and without post‐treatment relapse, so findings of this exploratory, post hoc analysis primarily should be considered hypothesis‐generating. The *P* values presented are nominal, rather than declarative, and may be affected by unaccounted‐for confounding variables. About one‐fifth of the overall population had no safety follow‐up and was excluded from the analysis; however, those lost to follow‐up had similar demographics and disease characteristics compared with those analyzed and therefore are less likely to be a source of systematic attrition bias. About one quarter of the analysis population had < 29 days of follow‐up, and about 31% had ≤ 60 days of follow‐up; given that the median time to relapse was 61 days, it is possible that some additional relapses occurred after follow‐up ended. Furthermore, participants with a < 28‐day follow‐up may have retained some protection against relapse during their limited follow‐up period since lymphocyte counts may not have returned to normal. The longer the follow‐up, the more relapses would be expected given the half‐life of ozanimod, particularly among participants who did not initiate another MS DMT. On the other hand, longer follow‐up time also might allow for relapsed participants to achieve a more complete recovery. Relatively strict prespecified criteria were used to identify relapses, which included a requirement for objective evidence of worsening based on EDSS or FS; therefore, we cannot rule out that these criteria resulted in undercounting the total number of relapses. However, relapses that are not associated with worsening on EDSS or FS scales are, by definition, relatively mild and are unlikely to contribute to evidence for rebound. Relapse severity and recovery were investigator‐determined, and set definitions were not provided to investigators to standardize assessments, resulting in some degree of subjectivity. Finally, the absence of MRI data at post‐treatment relapse limits the analysis to clinical disease activity. Nonetheless, our analysis is derived from a large clinical trial, and therefore, the results stem from high‐quality systematic assessments.

In conclusion, in DAYBREAK, no cases of post‐treatment relapse were found to have a pattern suggestive of a rebound effect. Although the lack of a harmonizing definition of rebound and varying methodologies makes it difficult to compare rates between studies, based on the available literature, the return of disease activity after permanent discontinuation of ozanimod appears to be milder and the occurrence of rebound less frequent relative to other MS DMTs that were associated with rebound effects following discontinuation, including natalizumab and fingolimod. Most DAYBREAK participants did not have MS relapses within 90 days of permanent ozanimod discontinuation. Post‐treatment relapses that did occur were predominantly 2–3 months after ozanimod discontinuation, mostly mild or moderate, and were usually followed by complete recovery. These data should be considered when discontinuing ozanimod (e.g., for family planning or other reasons). The vast majority of post‐treatment relapses were in untreated participants and were characteristic of the expected return of disease activity, which highlights the importance of counseling patients who are stable on ozanimod to continue treatment [1, 41]. In accordance with current guidelines, those who stop effective treatment for any reason should be encouraged to switch to another MS DMT promptly to minimize the risk of disease recurrence, especially if they had a high level of pretreatment disease activity [41, 42]. Patients who discontinue ozanimod and decline further treatment should be carefully monitored for recurrence and periodically re‐evaluate the need to reinitiate treatment [1].

## Author Contributions

Conception or design: R.G., K.W.S., R.B., J.A.C., G.C., E.K.H., J.K.S., H.D., C.‐Y.C., J.V.R., A.T., E.D., B.A.C.C. Data acquisition: K.W.S., J.A.C., G.C., E.K.H., B.A.C.C. Data analysis: H.D., C.‐Y.C. Data interpretation: R.G., K.W.S., R.B., J.A.C., G.C., E.K.H., J.K.S., H.D., C.‐Y.C., J.V.R., A.T., E.D., B.A.C.C. Review and approval of manuscript: R.G., K.W.S., R.B., J.A.C., G.C., E.K.H., J.K.S., H.D., C.‐Y.C., J.V.R., A.T., E.D., B.A.C.C.

## Funding

This work was supported by Bristol Myers Squibb.

## Conflicts of Interest

R.G. has received research support and speaker honoraria from Bristol Myers Squibb, which owns patent rights to ozanimod (a drug that was used in this study). K.W.S. has no conflicts to report. R.B. has been a consultant for Bristol Myers Squibb, which owns patent rights to ozanimod (a drug that was used in this study). J.A.C. has received personal compensation for consulting for Bristol Myers Squibb, which owns patent rights to ozanimod (a drug that was used in this study). G.C. has no conflicts to report. E.K.H. has no conflicts to report. J.K.S. is a former employee of Bristol Myers Squibb, which owns patent rights to ozanimod (a drug that was used in this study). H.D., C.‐Y.C., J.V.R., A.T., and E.D. are employees and/or shareholders of Bristol Myers Squibb, which owns patent rights to ozanimod (a drug that was used in this study). B.A.C.C. has no conflicts to report.

## Supporting information


**Data S1:** Supplementary Figures and Tables.

**Video S1:** acn370366‐sup‐0002‐VideoS1.mp4.

## Data Availability

BMS policy on data sharing may be found at https://www.bms.com/researchers‐and‐partners/independent‐research/data‐sharing‐request‐process.html.
