# Supplementary material for: Multiple Sclerosis Relapse Activity After Ozanimod Discontinuation in DAYBREAK Trial Participants
Source: Ann Clin Transl Neurol. 2026 Mar 27;13(10):2017–26. doi: 10.1002/acn3.70366 (PMC13394251; doi:10.1002/acn3.70366)
Supplement: Supplementary file 1 [file ACN3-13-2017-s002.docx]

**Supplementary Material**

**Supplemental Table S1. Previously Reported Definitions of Rebound After Discontinuation of Fingolimod**

| **Lead Author** | **Analysis Type** | **Definition** |
| --- | --- | --- |
| Vermersch et al, 2017^1^ | Post hoc analysis of phase 3 RCTs FREEDOMS and FREEDOMS II | Outliers with unexpectedly high disease activity consisting of any 1 of the following: (1) hospitalization due to a relapse, (2) relapse rated severe by the study investigator, (3) relapse with incomplete recovery or unusual EDSS increase. Unusual EDSS increase was defined as ≥ 3 for participants with a prior EDSS score of 0; ≥ 2 for participants with a prior EDSS score of 1 to 5; and ≥ 1 for participants with a prior EDSS score > 5, with prior being the latest available EDSS either on treatment but close to the time of study drug discontinuation or up to 30 days after discontinuation). |
| Callens et al, 2022^2^ | Retrospective analysis of prospective data from patients at 6 MS centers in Western France who discontinued for family planning purposes | Group level: Statistically significant increase in median ARR the year after fingolimod cessation compared with during fingolimod; posttreatment ARR was also compared with ARR in the pretreatment period  Patient level: disease reactivation surpassing the patient’s individual pretreatment level |
| Hatcher et al, 2016^3^ | Retrospective analysis of electronic records from UCSF Multiple Sclerosis Center | Rebound syndrome defined as new severe neurologic symptoms with multiple new or enhancing lesions exceeding baseline activity |
| Frau et al, 2018^4^ | Retrospective analysis of MS patients treated at 14 Italian MS centers | Severe reactivation defined as relapse with an associated ≥ 2-point increase in EDSS or ≥ 2 relapses in the 6 months after fingolimod discontinuation. A severe reactivation was considered rebound only if the patient had never previously in their lifetime had such a high level of severe reaction |
| Landi et al, 2022^5^ | Retrospective study of patients at 3 Italian MS Centers | Severe reactivation defined as any one of the following from 3–6 months after fingolimod discontinuation: (1) ≥ 2 relapses, (2) 1 relapse with worsening of ≥ 2 points on the EDSS, (3) presence of ≥ 3 GdE lesions on brain and/or spinal cord MRI |
| Maunula et al, 2024^6^ | Retrospective review of the Finnish MS register | Rebound defined as the following: (1) the most severe relapse in the patient’s history and an increase of ≥ 2 points on the EDSS during relapse occurring within 6 months of discontinuing fingolimod or (2) ≥ 1 relapse within 6 months of fingolimod discontinuation, with that being the patient’s highest lifetime relapse rate |
| Uygunoglu et al, 2018^7^ | Review of prospectively recorded data during and after fingolimod use from a single MS clinic at Istanbul University, Istanbul, Turkey | Severe disease reactivation defined as meeting all of the following: (1) > 5 GdE lesions and/or tumefactive demyelinating lesion on MRI, (2) clinical severity of the disease (evidenced by MRI) greater than the previous fingolimod treatment, requiring at least 7–10 days of intravenous methylprednisolone and/or plasma exchange, and (3) an increase of ≥ 1 point on the EDSS |
| Goncuoglu et al, 2021^8^ | Retrospective database analysis of patients treated with fingolimod at Hacettepe University Hospitals, Ankara, Turkey | Rebound defined as a new and unexpected severe relapse with ≥ 1 GdE lesion within 3 months of fingolimod discontinuation |
| Sepúlveda et al, 2020^9^ | Retrospective analysis of patients at the Hospital Clínic of Barcelona, Barcelona, Spain, who discontinued fingolimod for family planning purposes | Rebound defined as new severe neurological symptoms after ceasing fingolimod treatment, and the development of multiple new or enhancing lesions exceeding baseline activity |

ARR, annualized relapse rate; EDSS, Expanded Disability Status Scale; GdE, gadolinium-enhancing; ICU, intensive care unit; MRI, magnetic resonance imaging; MS, multiple sclerosis; RCT, randomized controlled trial.

**Supplemental Table S2. Baseline Demographic and Disease Characteristics for Those Included vs Those Excluded From the Analysis of Relapse After Ozanimod Discontinuation**

| **Participant characteristics** | **Analysis Population^a^**  **(n = 1679)** | **Participants Excluded from Analysis^b^  (n = 815)** |
| --- | --- | --- |
| Age at OLE consent, year, mean (SE) [range] | 36.7 (0.22) [19‒57] | 39.7 (0.33) [19‒57] |
| Sex, female, n (%) | 1120 (66.7) | 548 (67.2) |
| Race, White, n (%) | 1672 (99.6) | 802 (98.4) |
| Region, Eastern Europe, n (%) | 1577 (93.9) | 669 (82.1) |
| BMI ≥ 30 kg/m^2^, n (%) | 152 (9.1) | 124 (15.2) |
| Active smoker at OLE baseline, n (%) | 342 (20.4)^c^ | 175 (21.5) |
| MS DMT use prior to parent trial enrollment, n (%) | 464 (27.6) | 242 (29.7) |
| EDSS at OLE baseline, median (range), points | 2.5 (0.0–7.5) | 2.0 (0.0‒7.0) |
| Age at MS symptom onset, years, mean (SE) [range] | 28.6 (0.21) [9–53]^d^ | 31.3 (0.31) [12‒54]^e^ |
| Age at MS diagnosis, years, mean (SE) [range] | 31.8 (0.22) [13‒55]^d^ | 34.3 (0.32) [15‒55]^e^ |
| Number of relapses in 12 months before parent trial screening, mean (SE) [range] | 1.3 (0.02) [0‒5] | 1.2 (0.02) [0‒5] |
| Number of relapses in 24 months before parent trial screening, mean (SE) [range] | 1.8 (0.02) [1‒14] | 1.6 (0.03) [0‒5] |
| GdE lesion count at parent trial baseline, mean (SE) [range] | 1.8 (0.08) [0‒26]^f^ | 1.2 (0.09) [0‒18]^e^ |
| T2 lesion count at parent trial baseline, mean (SE) [range] | 53.7 (0.90) [0‒222]^f^ | 46.1 (1.19 [0‒219]^e^ |
| Number of relapses during phase 2 or 3 parent trial, mean (SE) [range] | 0.4 (0.02) [0‒7]^d^ | 0.3 (0.02) [0‒5]^e^ |
| Number of relapses during treatment in the OLE, mean (SE) [range] | 0.8 (0.04) [0‒11] | 0.4 (0.03) [0‒10] |
| Participants with relapse in the 12 months prior to ozanimod discontinuation, n (%) | 247 (14.7) | 47 (5.8) |

^a^The analysis population for assessment of posttreatment relapse consisted of DAYBREAK participants with ≥ 1 day of posttreatment safety follow-up who did not transition to commercial ozanimod within the 90-day safety follow-up period.
^b^Of the 2494 participants in the overall DAYBREAK population, 815 were excluded from the analysis of posttreatment relapse because they had no protocol-required safety follow-up (n = 523) and/or had initiated commercial ozanimod (n = 659).
^c^n = 1674.

^d^n = 1672.
^e^n = 802. ^f^n = 1671.

BMI, body mass index; DMT, disease-modifying therapy; EDSS, Expanded Disability Status Scale; GdE, gadolinium-enhancing; MS, multiple sclerosis, OLE, open-label extension; SE, standard error.

**SUPPLEMENTAL FIGURE S1.** Histogram for safety follow-up duration (days).

**SUPPLEMENTAL FIGURE S2.** Timing of posttreatment disease reactivation (relapse) (n = 55) after ozanimod discontinuation. ^a^Participants were not required to have more than 90 days of follow-up, but a ± 10-day window was permitted for the 90-day follow-up visit. One participant who relapsed after 93 days was within the protocol-defined ± 10 days follow-up window. The other, who relapsed after 141 days, was nonadherent to treatment due to COVID-19 travel restrictions. ^b^Excludes participants with <1 day of follow-up and those who switched to commercial ozanimod.

**SUPPLEMENTAL FIGURE S3.** Histogram of patients with non-zero change in EDSS during relapse following ozanimod in the DAYBREAK trial and during relapse in a historical control population of untreated patients with MS.^10^

**References**

1. Vermersch P, Radue EW, Putzki N, et al. A comparison of multiple sclerosis disease activity after discontinuation of fingolimod and placebo. Mult Scler J Exp Transl Clin 2017;3:2055217317730096.

2. Callens A, Leblanc S, Le Page E, et al. Disease reactivation after fingolimod cessation in multiple sclerosis patients with pregnancy desire: a retrospective study. Mult Scler Relat Disord 2022;66:104066.

3. Hatcher SE, Waubant E, Nourbakhsh B, et al. Rebound syndrome in patients with multiple aclerosis after cessation of fingolimod treatment. JAMA Neurol 2016;73:790-794.

4. Frau J, Sormani MP, Signori A, et al. Clinical activity after fingolimod cessation: disease reactivation or rebound? Eur J Neurol 2018;25:1270-1275.

5. Landi D, Signori A, Cellerino M, et al. What happens after fingolimod discontinuation? A multicentre real-life experience. J Neurol 2022;269:796-804.

6. Maunula A, Atula S, Laakso SM, et al. Frequency and risk factors of rebound after fingolimod discontinuation - A retrospective study. Mult Scler Relat Disord 2024;81:105134.

7. Uygunoglu U, Tutuncu M, Altintas A, et al. Factors predictive of severe multiple sclerosis disease reactivation after fingolimod cessation. Neurologist 2018;23:12-16.

8. Goncuoglu C, Tuncer A, Bayraktar-Ekincioglu A, et al. Factors associated with fingolimod rebound: a single center real-life experience. Mult Scler Relat Disord 2021;56:103278.

9. Sepúlveda M, Montejo C, Llufriu S, et al. Rebound of multiple sclerosis activity after fingolimod withdrawal due to planning pregnancy: Analysis of predisposing factors. Mult Scler Relat Disord 2020;38:101483.

10. Lublin FD, Baier M, Cutter G. Effect of relapses on development of residual deficit in multiple sclerosis. Neurology 2003;61:1528-1532.
